# Smokeless, not harmless: Understanding Naswar's cardiovascular risks in the northwestern Pakistan

**DOI:** 10.1016/j.pmedr.2025.102963

**Published:** 2025-01-13

**Authors:** Ihsanur Rahman, Fayaz Ahmad, Naveed Sadiq

**Affiliations:** Institute of Public Health and Social Sciences, Khyber Medical University, Peshawar, Pakistan

**Keywords:** Coronary artery disease (CAD), Smokeless tobacco (SLT), Naswar, Khyber Pakhtunkhwa, Pakistan

## Abstract

**Introduction:**

Tobacco use has a major impact on mortality from coronary artery disease (CAD). Naswar, a smokeless tobacco (SLT) product predominantly used in the northwestern part of Pakistan, has not been explored for its association with CAD. World Health Organization also expresses the need for further studies to thoroughly understand effects of different SLT products on CAD across various populations. This study explores the association between Naswar use and CAD in Peshawar, Pakistan.

**Methods:**

This case-control study recruited 112 CAD cases and 112 controls from tertiary care hospitals between July 2021 and August 2022. Consecutive sampling facilitated data collection through interviews using pre-structured questionnaire. Multivariable logistic regression analyses computed adjusted odds ratios for Naswar-CAD association. Prior ethical approval from the authors' institute, and informed consent from participants were obtained prior to conduction of study.

**Results:**

There were statistically significant differences in age distribution, income levels, house ownership, family history of heart disease, comorbidities, and Naswar usage between cases and controls. Multivariable logistic regression showed that Naswar users (OR: 2.56, 95 % CI: 1.08–6.10), Naswar dip time of five or more minutes (OR: 3.77, 95 %CI: 1.11–12.84), 20+ years of Naswar use (OR: 4.30, 95 %CI: 1.26–14.75), black Naswar (OR: 2.62, 95 %CI: 1.04–6.60), and spitting Naswar saliva (OR: 3.76, 95 %CI: 1.43–9.86) exhibited higher adjusted odds of CAD compared to non-users of Naswar.

**Conclusions:**

This study adds to the body of literature on significant association between Naswar use and CAD, emphasizing the necessity of regulating SLT products like Naswar to mitigate cardiovascular risks.

## Highlights

• Naswar users had higher odds of coronary artery disease.
• Naswar dip time of 5+ minutes increased coronary artery disease risk.
• 20+ years of Naswar use raised coronary artery disease odds.
• Black Naswar and its saliva spitting linked to higher coronary artery disease risk.
• Findings support regulating Naswar to reduce coronary artery disease risk.

## 1 Introduction

Coronary artery disease (CAD) is one of the most common non-communicable diseases not only globally but in low-and-middle-income countries like Pakistan as well. It is one of the major contributors to the global burden of morbidity and mortality (Roth et al., 2020). Globally, it has affected more than a half billion people, and was responsible for 20.5 million deaths in the year 2021 (Lindstrom et al., 2022), the highest among the Asian countries, where about 58 % deaths are attributed to CAD (Zhao, 2021). Even in Pakistan, CAD contributes to 16.49 % of all the deaths (Greenland, 2003; Khot et al., 2003; Vasan et al., 2005). Pakistan also exhibits the highest incidence of cardiovascular diseases globally, with a rate of 918.18 per 100,000 population compared to the global incidence of 684.33 per 100,000 (Metrics, 2019).

One of the leading causes of CAD worldwide and Pakistan is the use of tobacco (Saleheen et al., 2014). The prevalence of tobacco use in any form in Pakistan is 13.4 % (Basit et al., 2020). Tobacco is used primarily in four forms in Pakistan: cigarette smoking, Naswar dipping, paan, and gutka (Zakiullah et al., 2012). Naswar is a form of smokeless tobacco (SLT) which includes a mixture of various ingredients such as; lime, dried tobacco, sesame oil, ash, and water. For flavor, some ingredients such as menthol, indigo, and green cardamom are added to Naswar (Zakiullah et al., 2012). It is kept between cheek and gums (DrS et al., 2017). It is a popular, inexpensive, easily available, and a highly consumed product especially in the northwestern part of Pakistan, the Pashtun ethnic province of Khyber Pakhtunkhwa (Siddiqi et al., 2016). Naswar accounts for 60 % of the total tobacco consumed in Peshawar, which is the capital city of Khyber Pakhtunkhwa province (DrS et al., 2017).

Systematic reviews and meta-analysis studies have consistently linked SLT to various health issues, including cardiovascular diseases among others (Kawachi et al., 1993; McElduff et al., 1998; Infante-Rivard, 2003; Rahman et al., 2012; Rahman and Zaman, 2008; Ahmad et al., 2015). Specifically, research in South Asian countries has predominantly focused on the association between paan and gutka (as forms of SLT) and CAD, revealing a positive association (Titova et al., 2021; Huhtasaari et al., 1992). However, investigations into the effects of Naswar, another form of SLT, on CAD are limited (Janzon and Hedblad, 2009; Wennberg et al., 2007). Moreover, the global evidence regarding the relationship between SLT and CAD remains inconclusive (Vidyasagaran et al., 2016; Nahhas et al., 2022), emphasizing the need for further research to inform policies worldwide accurately. Thus, the primary objective of this study is to specifically examine the association between Naswar use and CAD.

## 2 Methods

### 2.1 Study design and setting

This case-control study was conducted from July 2021 to August 2022 in Peshawar, the capital of Khyber Pakhtunkhwa province of Pakistan. The cases and controls for this study were recruited from the tertiary care hospitals of the province. These hospitals have specialized cardiac care units for treating all sorts of cardiac diseases for all people residing in the province.

### 2.2 Study population

The study population consisted of the hospital-based cases and controls.

**Cases:** CAD patients admitted in the tertiary care hospitals' cardiac care specialty wards and diagnosed with CAD by a cardiologist were selected as cases. Admitted angina pectoris and myocardial infarction patients both were considered as cases of CAD for this study.

**Hospital controls:** Patients admitted to the medical wards of the same tertiary care hospitals were selected as controls. Controls were selected from the medical ward to ensure they were accessible and comparable to the cases in terms of the hospital environment and healthcare access. This helps to control for potential biases related to healthcare-seeking behavior and hospital admission criteria. All the controls had no medical history of CAD. Additionally, the controls consisted of patients with respiratory infections, gastrointestinal disorders, kidney infections, and minor surgical cases, unrelated to the CAD.

**Inclusion criteria:** Both the cases and controls were in the age group of 18–50 years, residents of the province, never smoked or not smoked in the past ten years, and willing to provide informed consent for the collection of data. The reason for not selecting the participants who quit smoking was that studies show that the risk of CAD decreases within an average of ten years after quitting smoking (Kawachi et al., 1993; McElduff et al., 1998; Infante-Rivard, 2003).

**Sample Size:** The sample size of the study was 224 participants (112 cases and 112 controls). Consecutive sampling method was used for the selection of participants. The sample size was calculated through Openepi software, using two-sided 95 %CI, 80 % power, and 1:1 case to control ratio. The proportion of SLT use among cases and controls were taken as 48.5 % and 30.2 % respectively from a Bangladeshi study (Rahman et al., 2012) as the prevalence of Naswar use among CAD and non-CAD were not known for Pakistan.

### 2.3 Ethical approval

This study adhered to institutional guidelines for protecting human subjects' safety and privacy. Ethical approval was obtained from the ethical review board of Khyber Medical University (KMU) (No. KMU/IPHSS/Ethics/2022/AB/073) and is on file. Permissions for data collection were granted by the relevant hospitals. Informed consent was obtained from each participant, and their identities were coded to ensure privacy and confidentiality.

### 2.4 Tools and data collection

Once the informed consent was taken from the eligible participants, the data were collected through an interview-based pre-structured questionnaire from both the cases and the controls. All the variables under this study were categorical in nature. The dependent variable was case or control. The independent variable in this study was the Naswar use (yes/no). The confounding variables were composed of socio-demographics, health-related risk factors, and lifestyle factors.

The socio-demographic variables included age group (18–29 years, 30–39 years, and above 39 years), sex (male/female), geographic location of the participants (central, northern, and southern regions of Khyber Pakhtunkhwa province), education (illiterate, three to nine years of education, 10–12 years of education, and 12+ years of education,), monthly income (no regular source, 17–113.4 United State Dollar (USD), 113.5–198.5 USD and 198.5+ USD), occupation (jobless, laborers, professional workers, businessmen and others), house ownership (rented/own), and presence of personal transport (no personal transport, personal car). The monthly income was captured in Pakistani currency, however, to provide an international perspective, the conversion rate for Pakistani currency was used to estimate the monthly income in the USD. The conversion rate from the middle of the study period, January 2022, was applied, where one USD equaled 176.3 Pakistani Rupees.

The health-related risk factors included family history of heart disease (yes/no), comorbidities (yes included hypertension, diabetes, and chronic obstructive pulmonary disease, and “no” included no other diseases that could contribute to CAD), and history of stress (yes/no).

The lifestyle variables included type of cooking fat used [oil (unsaturated fat), ghee (saturated fat), both], type of food mostly consumed (meat, vegetables, mix), exposure to passive smoking (no exposure to passive smoking, one to two times per day, more than three times per day), and exercise per week (never, daily, less than three times, three to five times).

Naswar user was defined as a person who daily used only Naswar at least for a year at the time of the study and before the occurrence of CAD. Similarly, a person who has quit Naswar for ten or more years or has never used in his lifetime was considered a non-Naswar user. Furthermore, we also investigated the other relevant information on Naswar use to assess dose-response relationship. In this regard, data on the type and years of Naswar use, Naswar unit dips and duration, whether the saliva was spit or swallowed were also collected.

**Data Analysis:** To assess association between the use of Naswar and CAD, multivariable logistic regression was applied to compute odds ratios, adjusted for various socio-demographic, health-related, and lifestyle factors that could potentially confound the relationship. The *p*-value of less than or equal to 0.05 was considered significant in this study. Data were analyzed through Statistical Package for the Social Sciences version 25. Percentages were computed for descriptive statistics for all the categorical variables.

## 3 Results

The study included 224 participants, 112 hospital cases and 112 hospital controls, ages ranged from 18 to 50 years. The mean age among cases was 43.65 ± 6.8 years while the mean age among controls was 31.73 ± 9.2 years. The overall mean was 37.69 ± 10.04 years.

### 3.1 Socio-demographic, health-related, and lifestyle characteristics of individuals

The Table 1 shows the association of various demographic, lifestyle, and health-related variables among cases and controls. Significant differences were observed in age distribution, with majority of cases falling in the 30–39 years (30.6 %) and 39+ years (77.1 %) age groups compared to controls where the majority belonged to the 18–29 years age bracket (89.5 %). Income levels showed a significant difference, with a higher proportion of cases reporting no regular income source (45.3 %) compared to controls. House ownership status also displayed a significant difference, with majority of cases residing in rented accommodations (33.3 %) compared to controls, who predominantly owned their homes (53.8 %). A significant association was found between family history of heart disease and case-control status, with a higher prevalence of family history among cases (63.6 %) compared to controls (36.4 %). Presence of comorbidities was significantly higher among cases (65.1 %) compared to controls (34.9 %). Notable differences were also observed in Naswar usage, with a higher proportion of cases reporting Naswar usage (60.2 %) compared to controls (39.8 %).

**Table 1:** Distribution of Socio-Demographic, Health-Related, Lifestyle, and Naswar-Related Variables among Cases and Controls in Adults Aged 18–50 Years in Northwestern Pakistan from Jul. 2021 - Aug. 2022 (*N* = 224).

Variables	Cases% (*n* = 112)	Controls% (n = 112)	Total% (N = 224)	*P* Value
Socio-Demographic Variables				
Age Group§				
18–29 Years	10.5	89.5	25.4	0.00
30–39 Years	30.6	69.4	21.9	
39+ Years	77.1	22.9	52.7	
Sex				
Male	49.7	50.3	87.9	0.84
Female	51.9	48.1	12.1	
Geographic Location of Participants				
Central Region	50.8	49.2	54.5	0.42
Northern Region	53.8	46.2	29.0	
Southern Region	40.5	59.5	16.5	
Years of Education				
Illiterate	51.6	48.4	27.7	0.30
3–9 Years	47.1	52.9	15.2	
10–12 Years	56.0	44.0	37.5	
12+ Years	38.6	61.4	19.6	
Monthly income (in USD)				
No Regular Source	45.3	54.7	23.7	0.00
17–113.4	34.7	65.3	32.1	
113.5–198.5	56.1	43.9	18.3	
198.5+	69.0	31.0	25.9	
Occupation				
Jobless	43.5	56.5	20.5	0.46
Manual Labour	57.9	42.1	25.4	
Professionals	41.7	58.3	16.1	
Businessman	54.5	45.5	19.6	
Others	48.8	51.2	18.3	
House Ownership§				
Rented	33.3	66.7	18.8	0.02
Own	53.8	46.2	81.3	
Presence of Personal Transport				
Yes	56.5	43.5	30.8	0.19
No	47.1	52.9	69.2	
Health-Related Variables				
Family History of Heart Disease§				
Yes	63.6	36.4	39.3	0.00
No	41.2	58.8	60.7	
Comorbidities§				
Yes	65.1	34.9	48.7	0.00
No	35.7	64.3	51.3	
History of Stress				
Yes	53.8	46.2	52.2	0.23
No	45.8	54.2	47.8	
Lifestyle Variables				
Type of Cooking Fat Used§				
Oil (Unsaturated Fat)	65.1	34.9	19.2	0.03
Ghee (Saturated Fat)	41.5	58.5	42.0	
Both	51.7	48.3	38.8	
Type of Food Mostly Consumed				
Meat	36.0	64.0	11.2	0.32
Vegetables	50.0	50.0	20.5	
Mix of both Meat & Vegetables	52.3	47.7	68.3	
Exposure to Passive Smoking				
1–2 Times per Day	44.9	55.1	21.9	0.57
More than 3 Times per Day	56.1	43.9	18.3	
No Exposure to Passive Smoking	50	50	59.8	
Exercise per Week				
Daily	43.2	56.8	49.6	0.17
3–5 Times	66.7	33.3	9.4	
<3 Times	54.1	45.9	16.5	
Never	54.5	45.5	24.6	
Naswar-Related Variables				
Naswar Use§				
Yes	60.2	39.8	39.3	0.01
No	43.4	56.6	60.7	
Type of Naswar Used§				
Black	61.1	38.9	32.1	0.04
Green	56.3	43.8	7.1	
Non-Naswar User	43.4	56.6	60.7	
Naswar Dip Time (minutes) ^§^				
1–5	47.2	52.8	23.7	0.00
5+	80.0	20.0	15.6	
Non-Naswar User	43.4	56.6	60.7	
Naswar Units Dipped per Day§				
1–10	61.8	38.2	24.6	0.04
10+	57.6	42.4	14.7	
Non-Naswar User	43.4	56.6	60.7	
Naswar Saliva§				
Swallow	58.8	41.2	7.6	0.04
Spit	60.6	39.4	31.7	
Non-Naswar User	43.4	56.6	60.7	
No. of Years Naswar Use§				
0–10	38.1	61.9	9.3	0.00
11–20	40.7	59.3	12.1	
21+	85.0	15.0	17.9	
Non-Naswar User	43.4	56.6	60.7	

§ Significant variables based on chi-sq based p-value of ≤ 0.05.

Among Naswar users, notable differences were observed in various aspects of Naswar consumption. Firstly, cases showed a higher preference for black Naswar (61.1 %) compared to controls (38.9 %). Secondly, cases were more likely to have used Naswar for 21+ years (85.0 %) compared to controls (15.0 %). Additionally, significant disparities were found in Naswar usage time, with cases more frequently using Naswar for five or more minutes (80.0 %) compared to controls (20.0 %). The number of Naswar dips per day also differed significantly, with cases more often consuming ten or more dips (57.6 %) compared to controls (42.4 %). Lastly, more cases reported swallowing Naswar saliva (58.8 %) compared to controls (41.2 %).

### 3.2 The adjusted odds

The results of the multivariable logistic regression analysis in Table 2 showed several significant and non-significant associations between various demographic, lifestyle, and health-related factors and the CAD. Among the significant variables, Naswar use showed a positive association with the CAD, with Naswar users demonstrating 2.89 times increased odds (95 %CI: 1.21–6.88) compared to non-users after adjusting for all confounders. Age group also showed significant associations, with individuals aged 18–29 years (OR: 0.03, 95 %CI: 0.01–0.11) and 30–39 years (OR: 0.08, 95 %CI: 0.03–0.24) exhibiting decreased odds of the CAD compared to those aged 39 years and older after adjusting for all confounders.

**Table 2:** Adjusted Odds Ratios of Various Risk Factors of Coronary Artery Disease in relation to Naswar among Adults Aged 18–50 Years in Northwestern Pakistan from Jul. 2021 - Aug. 2022 (N = 224).

Variables	Adjusted OR	95 % CI
Naswar-Related Variable		
Naswar Use		
Yes	2.89§	1.21–6.88
No	1.00	
Socio-Demographic Variables		
Age Group		
18–29 Years	0.03§	0.01–0.11
30–39 Years	0.08§	0.03–0.24
39+ Years	1.00	
Sex		
Male	0.47	0.10–2.25
Female	1.00	
Geographic Location of Participants		
Northern Region	1.50	0.59–3.78
Southern Region	0.39	0.14–1.15
Central Region	1.00	
Years of Education		
3–9 Years	0.78	0.22–2.79
10–12 Years	2.96	0.93–9.46
12+ Years	0.36	0.09–1.51
Illiterate	1.00	
Monthly income (in USD)		
17–113.4	0.59	0.18–1.90
113.5–198.5	1.19	0.29–4.78
198.5+	1.39	0.33–5.93
No Regular Source	1.00	
Occupation		
Manual Labour	1.36	0.30–6.13
Professionals	1.00	0.19–5.41
Others	0.78	0.16–3.84
Businessman	0.81	0.16–3.96
Jobless	1.00	
House Ownership		
Own	1.84	0.66–5.19
Rented	1.00	
Presence of Personal Transport		
Yes	2.15	0.73–6.35
No	1.00	
Health-Related Variables		
Family History of Heart Disease		
Yes	1.87	0.77–4.53
No	1.00	
Comorbidities		
Yes	1.81	0.77–4.28
No	1.00	
Lifestyle Variables		
History of Stress		
Yes	1.13	0.47–2.68
No	1.00	
Type of Cooking Fat Used		
Ghee (Saturated Fat)	0.33	0.11–1.00
Both	0.35	0.12–1.02
Oil (Unsaturated Fat)	1.00	
Type of Mostly Consumed Food		
Meat	0.56	0.12–2.53
Mix of both Meat & Vegetables	0.49	0.16–1.48
Vegetables	1.00	
Exposure to Passive Smoking		
1–2 Time per Day	0.68	0.23–2.02
More than 3 Times per Day	0.46	0.15–1.43
No exposure to Passive Smoking	1.00	
Exercise per Week		
3–5 Times	0.74	0.14–3.83
<3 Times	1.64	0.46–5.91
Daily	0.51	0.18–1.41
Never	1.00	

1.00 indicate Referent Level.

§ Significant ORs when the 95 %CI excludes “1”.

Regarding non-significant associations, variables such as gender, geographic location, education level, monthly income, occupation, house ownership, family history of heart disease, comorbidities, history of stress, cooking fat preference, predominant food consumption, frequency of exposure to passive smoking, and frequency of exercise per week did not show statistically significant associations with the CAD.

The mean age of cases was significantly higher than that of controls. Therefore, we examined potential interactions between age-group and Naswar use. After incorporating an interaction term for age and Naswar use, the results showed that the interaction was not statistically significant. As a result, we excluded the interaction term from the primary analysis. Details of the adjusted odds with the interaction term can be found in Supplementary Table S1.

Association of Different Factors of Naswar with CAD.

The Table 3 shows the associations between various aspects of Naswar use and the CAD, highlighting the importance of considering these factors in further research and intervention strategies.

• Naswar Use: Naswar users exhibited significantly higher odds of the CAD compared to non-users in both unadjusted (OR: 1.98, 95 %CI: 1.15–3.41) and adjusted models (OR: 2.89, 95 %CI: 1.21–6.88).
• Naswar Dips Duration in Mouth: Individuals using Naswar for five or more minutes had significantly higher odds of the CAD compared to those using it for one to five minutes, as indicated by both unadjusted (OR: 5.22, 95 %CI: 2.13–12.76) and adjusted models (OR: 2.53, 95 %CI: 0.91–7.02).
• Naswar Unit Dips per Day: While the unadjusted model showed significantly higher odds of the CAD for individuals consuming one to ten dips per day (OR: 2.11, 95 %CI: 1.11–4.01), this association was not statistically significant in the adjusted model.
• Years of Naswar Use: Individuals with 21+ years of Naswar use exhibited significantly higher odds of the CAD compared to non-users, both in unadjusted (OR: 7.40, 95 %CI: 2.91–18.78) and adjusted models (OR: 4.71, 95 %CI: 1.36–16.28).
• Types of Naswar Use: Using different varieties of black Naswar was associated with significantly higher odds of the CAD compared to non-users, as indicated by both unadjusted (OR: 2.05, 95 %CI: 1.15–3.67) and adjusted models (OR: 3.03, 95 %CI: 1.21–7.54).
• Naswar Saliva: Individuals who spat Naswar saliva had significantly higher odds of the CAD compared to non-users, as shown in both unadjusted (OR: 2.00, 95 %CI: 1.18–3.60) and adjusted models (OR: 4.37, 95 %CI: 1.66–11.54).

**Table 3:** Unadjusted and Adjusted Odds Ratios for Naswar Use-Related Variables in Assessing Risk of Coronary Artery Disease among Adults Aged 18–50 Years in Northwestern Pakistan from Jul. 2021 - Aug. 2022 (N = 224).

Naswar Related Variables	Unadjusted OR	95 % CI	Adjusted OR⁎	95 % CI
Naswar Use				
Yes	1.98§	1.15–3.41	2.89§	1.21-6.88
No	1.00			
Naswar Dip Time (minutes)				
1–5	1.17	0.62–2.20	1.99	0.71–5.54
5+	5.22§	2.13-12.76	3.77§	1.11-12.84
Non-Naswar User	1.00			
Naswar Units Dipped per Day				
1–10	2.11§	1.11–4.01	2.34	0.84–6.56
10+	1.77	0.82–3.82	2.94	0.88–9.80
Non-Naswar User	1.00			
No. of Years Naswar Use				
0–10	0.80	0.31–2.06	2.98	0.67–13.27
11–20	0.90	0.39–2.08	1.23	0.36–4.27
21+	7.40§	2.91-18.78	4.30§	1.26-14.75
Non-Naswar User	1.00			
Types of Naswar Used				
Black	2.05§	1.15–3.67	2.62§	1.04-6.60
Green	1.678	0.59–4.77	2.32	0.44–12.32
Non-Naswar User	1.00			
Naswar saliva				
Swallow	1.86	0.67–5.19	0.67	0.15–3.00
Spit	2.00§	1.18-3.60	3.76§	1.43-9.86
Non-Naswar User	1.00			

1.00 indicate referent level.

⁎ Adjusted for socio-demographics variables (age group, sex, geographical location, years of education, monthly income, occupation, house ownership, and presence of personal transport), health-related variables (family history of heart disease, and comorbidities), and lifestyle related variables (history of stress, types of cooking fats used, types of mostly consumed food, exposure to passive smoking, and exercise per week).

§ Significant ORs when the 95 %CI excludes “1”.

## 4 Discussion

This hospital-based case control study was conducted in Pakistan to assess the association between Naswar use and the risk of CAD among adult population. The findings reveal that the use of Naswar is significantly associated with CAD. A positive association with CAD was also observed with the dose response relationship in terms of dip time, number of years of Naswar use, the type of Naswar used, and Naswar's saliva being swallowed or spitted.

The finding of positive association between Naswar and CAD is similar to the studies that explored the relationship between other forms of SLT and cardiovascular diseases (Rahman and Zaman, 2008; Ahmad et al., 2015; Titova et al., 2021; Huhtasaari et al., 1992). The odds of CAD among other forms of SLT (OR: 1.40, 95 %CI: 1.01–1.95) users in the meta-analysis study of the South Asian countries were comparable to our study findings (Vidyasagaran et al., 2016). However, a variation in the odds of CAD among Naswar users in our study and other specific SLT products used around the globe was observed when compared to other studies. For instance, the odds of CAD among Naswar users (OR: 2.89; 95 %CI: 1.21–6.88) were lower than the study published on the Bangladeshi population (OR: 4.0; 95 %CI: 2.0–8.1). This variation could be attributed to differences in the SLT products used elsewhere. In our study, the SLT product is Naswar (a combination of lime, dried tobacco, sesame oil, ash, and water), while in Bangladesh, it's a combination of betel nuts with quid and dried tobacco leaf (Rahman and Zaman, 2008).

When comparing our findings with the Bangladeshi study (Rahman and Zaman, 2008), a similar age distribution was observed among cases in both studies, with the majority of participants in the 39+ age group (73.9 % in the Bangladeshi study vs. 77.1 % in our study). However, the age distribution of controls differed significantly. In the Bangladeshi study, 36.2 % of controls were aged 30–39, whereas in our study, 89.5 % of controls were in the 18–29 age group.

Regarding sex distribution, the Bangladeshi study (Rahman and Zaman, 2008) reported a higher proportion of males among both cases and controls, whereas our study showed a nearly equal distribution of sexes in both groups. Family history of heart disease and comorbidities such as diabetes were insignificant predictors in both studies; however, hypertension was significantly higher in the case group in our study.

In adjusted analyses, both age group and sex were significant predictors in the Bangladeshi study (Rahman and Zaman, 2008), whereas only age group was significant in ours. Notably, the Bangladeshi study adjusted for confounders based solely on significance in chi-square tests, while we accounted for all known potential confounders, irrespective of their chi-square significance.

The hazard risk for cardiovascular diseases among snus users in Swedish (Titova et al., 2021) and American (Nahhas et al., 2022) populations was not significant. Demographic differences between our study population and those in the Swedish and American studies were evident. The Swedish population studied had an average age of 65.5 years, compared to a mean age of 37 years in our population. While the Swedish study predominantly involved males (93.4 %), our study also included a high proportion of males (87.9 %). Similarly, the American study focused exclusively on males with a mean age of 58.9 years. Despite higher risks of heart diseases in older populations, neither Swedish nor American studies reported an increased risk of cardiovascular disease with smokeless tobacco (SLT) use. This complies with findings from a systematic review by Vidyasagaran et al. (Vidyasagaran et al., 2016), which identified a significant increase in cardiovascular risk among SLT users in Asia but not in Europe.

We grouped all comorbidities into a single variable, while comparison studies reported them separately. In Swedish studies, Wennberg et al. (Wennberg et al., 2007) reported 34.3 % of participants with hypertension and 6.1 % with diabetes among cases. Janzon et al. (Janzon and Hedblad, 2009) reported higher proportions of hypertension (∼65 %) and diabetes (3.9 %), while Titova et al. (Titova et al., 2021) reported 38.6 % with hypertension and 9.8 % with diabetes. In contrast, Nahhas (Nahhas et al., 2022) reported 50.7 % with hypertension and 27.6 % with diabetes. Our study reported 48.7 % for all comorbidities combined.

Regarding literacy levels, no significant association was found between cases and controls in our study. However, Wennberg et al. (Wennberg et al., 2007) observed predominantly low literacy levels among cases (59.2 %). Titova et al. (Titova et al., 2021) reported 76.8 % of participants having high school or below education, while Nahhas noted 41.1 % of participants were high school graduates or below (Nahhas et al., 2022).

We note that there are geographical variations in the association of SLT and heart diseases in our study and studies conducted in western part of the world. These findings emphasize the need for comprehensive policies and regulations regarding SLT products, including Naswar, to mitigate their adverse effects on cardiovascular health. Further research is warranted to explore the underlying mechanisms of SLT-induced cardiovascular damage, evaluate the effectiveness of regulatory interventions, and develop targeted prevention and cessation strategies for SLT users, especially Naswar. The future research may also study the effects of various ingredients, used in the preparation of SLT including Naswar, on cardiovascular health. By addressing these gaps in knowledge, public health efforts can be better informed and tailored to reduce the burden of CAD associated with SLT use.

The study is limited by the fact that a hospital-based sample was used that may not be a representative of a community (Wacholder and Hartge, 2014). This may have introduced some bias in the assessment of relationship between Naswar and CAD (Wacholder and Hartge, 2014). However, there are some studies that conclude that hospital controls could be as good as the population controls and in some cases hospital controls may be preferred (Infante-Rivard, 2003; Ruano-Ravina et al., 2008; Li et al., 2011). To overcome this limitation, we suggest that a study with a population control may be undertaken to assess the association of Naswar and CAD. Another limitation of our study was that we did not collect data on medical or surgical conditions of the control group, therefore, we could not perform additional analyses to check for any indirect associations between the control conditions and CAD.

In a nutshell, there seems to be an association between Naswar and CAD. The chemicals like nicotine, nitrates, and oxidative levels of the Naswar, together with lifestyle and Naswar habits may contribute to the development of CAD. For any policy regarding the SLT, it may be necessary to examine the constituents of the SLT for their deteriorative impact on cardiovascular health. Due to high taxes and increased awareness, people switch from tobacco smoking to Naswar use, as it is available on a reasonable and affordable price in Pakistan (O'Connor, 2012). Similar to smoking, policies are imperative for Naswar use. Majority of the people are aware of the effects of tobacco smoking on heart and lungs (Siahpush, 2006), however, there is limited awareness about the harmful effects of Naswar use, primarily due to little research on it (Naswar). Many studies have recommended campaigns on broad level for tobacco control (Perry et al., 1992; Farrelly, 2003; Friend, 2002), such as strengthening health systems to ensure a tobacco-free environment. The findings of this study could be extended to countries where Naswar is frequently used.

## Funding

No funding was received from any source at any stage of this research/study.

## Declaration of generative AI and AI-assisted technologies in the writing process during revisions

During the revision of this work the authors used Microsoft Co-Pilot to improve language and readability in the revised sections. After using this tool/service, the authors reviewed and edited the content as needed and take full responsibility for the content of the publication.

## CRediT authorship contribution statement

**Ihsanur Rahman:** Writing – review & editing, Writing – original draft, Software, Methodology, Investigation, Data curation, Conceptualization. **Fayaz Ahmad:** Writing – review & editing, Writing – original draft, Supervision, Software, Methodology, Data curation, Conceptualization. **Naveed Sadiq:** Writing – review & editing, Writing – original draft, Validation, Supervision, Software, Methodology, Data curation, Conceptualization.

## Declaration of competing interest

The authors declare that they have no known competing financial interests or personal relationships that could have appeared to influence the work reported in this paper.

## Data Availability

Data will be made available on request.

## Appendix A Supplementary data

Adjusted Odds Ratios for Coronary Artery Disease Risk, Including Interaction of Age Group and Naswar Use, in Adults Aged 18-50 Years in Northwestern Pakistan from Jul. 2021 - Aug. 2022 Image 1
