# Supplementary material for: Smokeless, not harmless: Understanding Naswar's cardiovascular risks in the northwestern Pakistan
Source: Prev Med Rep. 2025 Jan 13;50:102963. doi: 10.1016/j.pmedr.2025.102963 (PMC11783110; doi:10.1016/j.pmedr.2025.102963)
Supplement: Supplementary file 1 — Adjusted Odds Ratios for Coronary Artery Disease Risk, Including Interaction of Age Group and Naswar Use, in Adults Aged 18-50 Years in Northwestern Pakistan from Jul. 2021 - Aug. 2022 [file mmc1.docx]

Supplementary Table S1: Adjusted Odds Ratios for Coronary Artery Disease Risk, Including Interaction of Age Group and Naswar Use, in Adults Aged 18-50 Years in Northwestern Pakistan from Jul. 2021 - Aug. 2022

| Variables | Adjusted OR | 95% CI |
| --- | --- | --- |
| Naswar Use | | |
| Yes | 3.35^§^ | 1.06-10.64 |
| No | 1.00 | |
| Socio-Demographic Variables | | |
| Age Group | | |
| 18-29 Years | 0.02^§^ | 0.00-0.10 |
| 30-39 Years | 0.10^§^ | 0.03-0.35 |
| 39+ Years | 1.00 | |
| Sex | | |
| Male | 0.43 | 0.09-2.18 |
| Female | 1.00 | |
| Geographical Location of Participants | | |
| Northern Region | 1.37 | 0.54-3.49 |
| Southern Region | 0.44 | 0.15-1.31 |
| Central Region | 1.00 | |
| Years of Education | | |
| 3-9 Years | 0.76 | 0.21-2.67 |
| 10-12 Years | 2.50 | 0.77-8.17 |
| 12+ Years | 0.36 | 0.09-1.43 |
| Illiterate | 1.00 | |
| Monthly income (in USD) | | |
| 17-113.4 | 0.55 | 0.17-1.83 |
| 113.5-198.5 | 1.30 | 0.32-5.24 |
| 198.5+ | 1.29 | 0.31-5.39 |
| No Regular Source | 1.00 | |
| Occupation | | |
| Manual Labour | 1.17 | 0.24-5.58 |
| Professionals | 1.02 | 0.18-5.69 |
| Others | 0.79 | 0.15-4.08 |
| Businessman | 0.98 | 0.19-5.11 |
| Jobless | 1.00 | |
| House Ownership | | |
| Own | 2.18 | 0.75-6.32 |
| Rented | 1.00 | |
| Presence of Personal Transport | | |
| Yes | 1.70 | 0.58-4.99 |
| No | 1.00 | |
| Health-Related Variables | | |
| Family History of Heart Disease | | |
| Yes | 2.20 | 0.91-5.33 |
| No | 1.00 | |
| Comorbidities | | |
| Yes | 0.45 | 0.18-1.11 |
| No | 1.00 | |
| Lifestyle Variables | | |
| History of stress | | |
| Yes | 1.46 | 0.61-3.52 |
| No | 1.00 | |
| Type of Cooking Fat Used | | |
| Ghee (Saturated Fat) | 0.35 | 0.11-1.11 |
| Both | 0.36 | 0.12-1.07 |
| Oil (Unsaturated Fat) | 1.00 | |
| Type of Mostly Consumed Food | | |
| Meat | 0.61 | 0.13-2.88 |
| Mix of both Meat & Vegetables | 0.63 | 0.21-1.92 |
| Vegetables | 1.00 | |
| Exposure to Passive Smoking | | |
| 1-2 Time per Day | 0.54 | 0.18-1.60 |
| More than 3 Times per Day | 0.43 | 0.14-1.38 |
| No exposure to Passive Smoking | 1.00 | |
| Exercise per Week | | |
| 3-5 Times | 0.64 | 0.12-3.35 |
| <3 Times | 1.79 | 0.49-6.59 |
| Daily | 0.42 | 0.15-1.16 |
| Never | 1.00 | |
| Interaction (Naswar Use * Age Group) |  |  |
| Yes (Naswar User) * 18-29 Years | 2.07 | 0.20-21.30 |
| Yes (Naswar User) * 30-39 Years | 0.38 | 0.06-2.31 |
| No (Non-User of Naswar) * 39+ Years | 1.00 | |

^§^*Significant ORs when the 95%CI excludes “1”*

*1.00 indicate referent level*
